# New Era of Electroceuticals: Clinically Driven Smart Implantable Electronic Devices Moving towards Precision Therapy

**DOI:** 10.3390/mi13020161

**Published:** 2022-01-22

**Authors:** RaviPrakash Magisetty, Sung-Min Park

**Affiliations:** 1 Department of Convergence IT Engineering, Pohang University of Science and Technology (POSTECH), Pohang 37673, Korea; magiravi4e@gmail.com; 2 Department of Electrical Engineering, Pohang University of Science and Technology (POSTECH), Pohang 37673, Korea; 3 Department of Mechanical Engineering, Pohang University of Science and Technology (POSTECH), Pohang 37673, Korea

**Keywords:** electroceuticals, miniaturization, wireless power transfer, low voltage, biocompatibility

## Abstract

In the name of electroceuticals, bioelectronic devices have transformed and become essential for dealing with all physiological responses. This significant advancement is attributable to its interdisciplinary nature from engineering and sciences and also the progress in micro and nanotechnologies. Undoubtedly, in the future, bioelectronics would lead in such a way that diagnosing and treating patients’ diseases is more efficient. In this context, we have reviewed the current advancement of implantable medical electronics (electroceuticals) with their immense potential advantages. Specifically, the article discusses pacemakers, neural stimulation, artificial retinae, and vagus nerve stimulation, their micro/nanoscale features, and material aspects as value addition. Over the past years, most researchers have only focused on the electroceuticals metamorphically transforming from a concept to a device stage to positively impact the therapeutic outcomes. Herein, the article discusses the smart implants’ development challenges and opportunities, electromagnetic field effects, and their potential consequences, which will be useful for developing a reliable and qualified smart electroceutical implant for targeted clinical use. Finally, this review article highlights the importance of wirelessly supplying the necessary power and wirelessly triggering functional electronic circuits with ultra-low power consumption and multi-functional advantages such as monitoring and treating the disease in real-time.

## 1. Introduction

Bioelectronic devices have been used in several medical applications with many features and components [1]. The global market for active biomedical implants is expected to reach a compound annual growth rate of 8.5 % between 2021 and 2027 (forecast period), with a base value of 178.9 billion USD in 2020 [2]. This rapid growth is probably due to the patient’s clinical needs. From the device’s point of view, the transition may be due to device design and function, efficacy and safety, and adaptability. Several implantable bioelectronics were reported in the literature. However, stimulating cardiac tissues to generate artificial heartbeat, stimulating brain neurons for treating neurological disorders, stimulating action to create an artificial vision, and stimulating vagus nerve for enabling multi-organ functions have been gaining attention due to their remarkable benefits for people with various clinical problems [3,4,5,6,7,8,9,10,11,12,13,14]. Representative smart implants for these include a cardiac pacemaker, deep brain stimulator, vagus nerve stimulator, and artificial retina.

Fundamental biological bodies such as the heart and brain are central to the human body’s many complex intellectual and biological operations. The eye is a smart, intelligent, and complex biological machine that can help us visualize and perceive light. Furthermore, the vagus nerve consists of a heterogeneous group of neural fibers innervating numerous organs. Monitoring and treating abnormal conditions of these human organ systems are prime importance of medical interventions. Thus, pacemakers and defibrillator devices were successfully developed to treat cardiac arrhythmias; arrhythmia is an irregularity in a heartbeat [15]. Furthermore, a cardiovascular implantable device was introduced for managing the superior vena cava syndrome [6]. Epilepsy is a non-communicable neurological disorder due to abnormal electrical activity in the brain that needs to be treated to reduce psychological problems. Grids and strips-based devices were successfully developed to map epilepsy cortical cerebral activity [16,17]. Parkinson’s disease is another neurological disorder. Electrodes stimulate deep brain tissues to treat Parkinson’s disease [18]. Furthermore, neuro-prosthetics: a biological brain pacemaker as a living neuro-modulatory implant for improving parkinsonian symptoms [6] have been successfully introduced. Moreover, retinal prosthesis devices [9,19], microelectrode arrays for artificial retinal implants using liquid crystals [10], micro-biosensors for glucose, and L-glutamate monitoring in the retina [11], etc., have been successfully developed. Furthermore, vagus nerve stimulation for the treatment of epilepsy [11], for improving brain functions [12], and for stroke recovery [12], etc., have been introduced by Ben-Menachem et al., Kavakbasi et al., and Pruitt et al.

Researchers have also developed devices that could gain energy wirelessly and supply it to the implanted electronic circuit to trigger stimulations and other operations [7,8,9,10,19], such as a photovoltaic array-based artificial retina system [19] and self-powered devices to trigger impulsive responses to recover heartbeat [20,21,22,23,24,25,26,27,28]. Such a fully wireless-powered or self-powered integrated implant is a step forward in recovering normal biological activity and exhibits significant potential advantages [7,8,9,10,19]. It is possible to attain the test data without skin interference, reduce skin/device interaction, prevent repeated surgeries, monitor onsite and treat the diseases that could be possible, etc., [7,8,9,10,19]. However, the existing systems still have challenges and limitations [7,8,9,10]. For example, though the devices have bi-directional communication capabilities, they suffer from low transmitted power, probably due to low peak current at a predetermined resistance, signal-to-noise ratio, impedance mismatch, consequently low data transmission capability [20,29]. Depending on application need, the external transmitter device’s controller must be a simple electronic circuit that could handle the entire operation in an intelligent environment. The device must handle the whole operation from the sending end to the receiving end, the power and data, subsequent data modulation, demodulation, and power management such as amplification, filtering, protection, etc., [2,7,8,9,10,14]. From the point of the implantable device, i.e., receiver, the engineer/designer must consider all factors relevant to the device’s operational parameters, such as maximum and minimum operational currents and voltages, data rates. It is also a prime concern that the engineer/designer consider the safety and security of tissues surrounded by the implant device, vice versa, as a matter of the fact that a high-power consumption or high current or voltages of an implantable device increases the temperature of tissues causing damage by dissipating heat. When powering up the implanted electronic device, the factors (high current or voltages) also influence the high density per cubic centimeter of electromagnetic fields through the tissues. Therefore, apart from maintaining optimum power consumption, some features to ensure robust communication and secure stimulation are also desirable for efficiently monitoring and treating diseases.

In this context, we have summarized significant electroceuticals; pacemaker, neural stimulation, artificial retina, and vagus nerve stimulation. We have discussed smart implants development challenges and opportunities. Furthermore, micro and nanoscale features and material aspects, electromagnetic field effects on innovative biomedical implants, their potential consequences have been presented. The report highlights the advancement in micro/nanoscale fabrication technologies and their use in fabricating electroceuticals for targeted clinical needs. Finally, the importance of wireless power transfer and trigger-enabled functional electronic circuits with ultra-low power consumption has been discussed.

## 2. Needs for Next-Generation Electroceuticals: Intelligent Biomedical Implants

**Figure 1:**
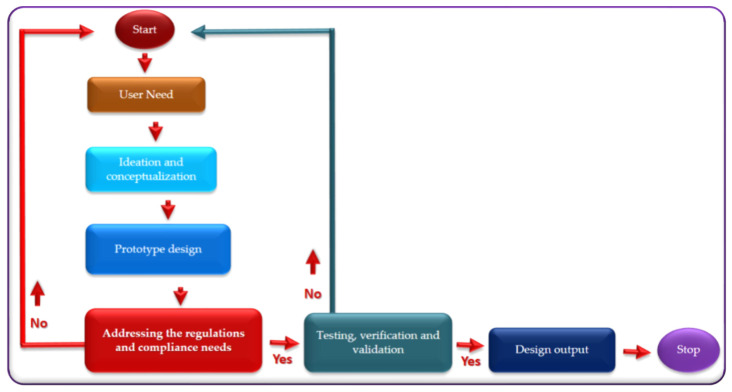
Electroceutical device development/fabrication process flow chart.

Electroceuticals are emerging as a smart tool to prevent and cure diseases and boost the tissue functions to heal itself. No doubt, electroceuticals will change our daily routines. However, risks should not be ignored. Still, when designing biomedical electronic devices or systems, several risk factors need to be taken care of to provide an excellent design to assist patients’ concerns efficiently. Electroceuticals must provide timely treatment and care without the patient’s internal infections such as pulmonary embolisms, post-operative hemorrhages, respiratory emergencies, etc. Importantly, when designing and fabricating implantable electroceutical devices, the device performance parameters under biological and environmental factors must be considered. Furthermore, during the initial stages of implantable device design, the engineer must consider the guidelines and regulations defined by the concerned authority or country. Most countries follow guidelines and rules prescribed by the food and drug administration (FDA) and the international electro technical commission (IEC) (IEC-60601-1 and IEC-60601-1-2) [30,31]. Good manufacturing practices are also needed for a qualified electroceutical design. Choosing quality materials among the material resources is also a part of skillful product design. Importantly, the product design needs to address the occurrence versus severity of harm. The development/fabrication of an electroceutical device must undergo the following steps: need-identification, conceptualization, prototype design, addressing the regulations, and compliance need (Figure 1). The detailed process flow diagram is illustrated in Figure 1.

Major phases in medical device development are; conceptualization, development, manufacturing, and labeling. These phases need to be reorganized if any of them affect the safety and performance of a medical device (Figure 1). For example, for improving vision, retinal neurons must be stimulated with specific impulsive responses at optimum intensity—similarly, a cardiac pacemaker must deliver a minute electrical impulse of a particular size and shape. Significant deviation from this may cause violation of safety and performance, leading to complications in patients’ health. Maybe more complex designs could impose several risk factors as user errors. Therefore, we must consider a simple concept to ensure the design parameters and performance characteristics do not inflict unwanted risks.

Electromagnetic interference has become a potential risk for electroceuticals. This interference problem has grown far more complicated mainly because of two reasons; the advancement in micro/nano miniaturization technologies, which enables smaller, even nanoscale devices with low operating current, voltage, and power. The second one is the tremendous growth in the source of radiofrequency energy. The electromagnetic interference negatively affects the device function/operation and introduces abnormalities during the device’s optimum operating conditions. Electromagnetic interference occurs when electromagnetic waves are emitted by one device and impede the normal operation of another device. Let us assume radio signals injected on an un-shielded electronic chip such as without a metallically isolated retinal implant device. The unshielded electronic chip would act as a detector, which demodulates the high-frequency (radio signal) to a low-frequency demodulated noise signal. These demodulated noise signals disturb an implanted device’s regular operation and create abnormalities that render an unconditional attack on the biological tissues [32].

According to WHO, static electric fields do not penetrate the living systems and only be able to induce currents on the surface of an organ (electrically active), so there are no rich enabled risks with these electric fields [33]. A magnetic reed switch exists in some pacemakers, which is closed by static magnetic fields. However, a strong static magnetic field could activate the magnetic reed switch leading to asynchronous pacing for the pacemaker and disabling tachycardia therapy in ICDs [34]. Low-frequency electromagnetic field interactions with the electronic circuits and the materials induce electromotive force triggered voltages and currents; the density of these electric currents and voltages depend on the intensity of the field. These induced signals could superimpose with the operating signals of an implanted device and cause either misleading recording information or introduce abnormal disturbance during the device’s operations. This field would also induce electric voltage at the connecting leads or in the electric circuit that leads to a malfunction or damage of the component. In the case of radiofrequency field exposure, the implantable device’s surface (conducting) acts as an antenna. It induces electromotive force rendered voltage spikes, thus disturbing the sensing or stimulation operations, etc.

Hence the federal food and drug administration (FDA) has introduced safety regulations, particularly for electroceutical concerning electromagnetic interference and device compatibility. According to the safety requirement, the electroceutical device must undergo an electromagnetic compatibility (EMC) test. The device must be validated (efficiency, accuracy, etc.) throughout the test. In detail, there should not be device resets, electronic component failures or damages, operation and programmable parameter alteration, error messages, patient’s false information [30,31]. The device’s EMC tests are emission tests such as electromagnetic interference (EMI) defined by the Comite International Special des Perturbations Radioelectriques (CISPR), radiation emission tests (RFI) determined by the Federal Communication Commission (FCC), other regulations stipulated by the Medicines and Healthcare products Regulatory Agency (MHRD) and European Union (EU) regulations. Furthermore, the American Society for Testing and Materials (ASTM) standard tests such as biocompatibility, implantable devices, materials, etc.

## 3. Smart Implants Development Challenges

Despite current progress in implantable bioelectronics such as electroceuticals, many challenges must be addressed to provide safe, precise, and targeted therapy. Many implanted bioelectronic devices are wired with battery-powered and bulky, which are invasive, causing discomfort to the patient and limiting their usage. As an alternative, Kim et al. succeeded in developing a wireless implantable bioelectronic device that can compete with traditional ones [35]. However, these kinds of devices also exhibit some challenges. Among them, the challenges that must be addressed are relevant to efficiency, biocompatibility, etc., [36,37,38]. How do these devices efficiently transfer energy inside of the body without any losses? What are the available sources of power? Can these power sources be compatible with the biological body and the communicated device located outside of the body? Do they adapt with the surrounding tissues or biological cells without creating any abnormalities? Can we miniaturize them as small as in nanoscale, or maybe more? Can we combine implanted device technology and nanotechnology so that the miniaturized implantable devices cannot hurt the surrounding biological tissues while efficiently treating the disease? Do they communicate securely with the external device? What sort of electronics components and feedback loops are needed to help the user control the device functionalities? What kind of software is necessary to control them remotely? How do we tackle these challenging questions to provide a better therapy is gaining attention? More recently, these challenges are gaining attention concerning pacemaker, neural stimulation, artificial retina, and vagus nerve stimulation-based device development, which is attributable to the importance of these implantable biomedical machines crucial for thinking, acting, and living well.

Designing an implantable wireless electroceutical device with its receiver antennae or an inductive coil-based receiver system is still bulky due to its limitation in miniaturization capability-enabled functionality. To operate an electroceutical device, a few to several hundred microwatts of energy is required. The optimized design (length, height, and width while maintaining material functional parameters) is needed to deliver enough power to handle designated therapeutic operations. Thus, a considerable challenge must be addressed when miniaturizing the wireless power transfer system [37,38,39]. This challenge can be overcome with the help of advanced materials, nanotechnologies-enabled functional components, and electromagnetic simulation tools that optimize designs.

Though current technologies are capable of triggering activities of respective tissues, miniaturized electroceutical devices capable of both monitoring and treating functionality integrated into a single device are of industrialists’ and biomedical engineers’ current interest [35,40,41]. There is significant interest in developing wireless devices that could monitor, record, and treat neural activity. For example, for better vision, the artificial retina system could stimulate the retinal neurons by measuring their abnormal activity [35,41,42]. Similarly, a pacemaker system could deliver impulsive responses by understanding the abnormal activity of the heart through the implantable electrocardiograph monitoring device [35,41,42]. Although the micro/nanoscale technologies could help in miniaturizing wireless power transfer receivers as a part of an intelligent system, developing multifunctional advantages in an integrated electronic chip by considering all relevant factors may be a challenging task. A great challenge is developing an integrated system with sophisticated electronic circuits that could help interface, interact, communicate with the outer environment and the systems, and perform multiple tasks. Integrating all these into a fabrication sequence, especially integration with other devices and small electronic components to make a miniaturized system, is a significant challenge. A heterogeneous wireless communication system with further attention to low power consumption is difficult.

A great challenge is developing miniaturized wireless power transfer circuits with long-term stability for biomedical applications [43]. Using an additional circuit that is needed to rectify the generated AC output voltage is challenging because the circuit reduces the total system efficiency [43]. The main challenge is their large size because of integrating several components for the desired operation and limited ability to incorporate power delivery [43]. The challenges remain in terms of material innovation, device fabrication, and circuit design architecture [44]. Although the current micro/nanoscale technologies enable the development of biomedical electronics, the performance of the end component/device is low concerning the efficiency to that of the clinical condition and the material compatibility to a respective biological environment [45]. This must be addressed more effectively and efficiently and can be overcome with precise and accurate electronic devices, conductive interconnects, and fluidic manifolds at the micro/nanoscale dimensions to enable low power and voltage operation capability enabled by advanced micro/nanofabrication techniques.

Developing a single dedicated sensor for sensing and monitoring all biological functions of multiple parameters is hard and challenging. For example, the sensor that can sense breath under different atmospheric conditions or a single sensor that can sense multiple physical functionalities such as motion, pressure, heart rhythms, etc., are challenging tasks. In addition to sensing biochemical and biophysical functionalities, sensing optical and electrical functionalities from brain neurons or neural retina are complex. Multifunctional materials, including copper-phthalocyanine, carbon, graphene, etc., could be a thought of potential materials to develop lab-on-a-chip devices capable of sensing both physical and biochemical parameters in response to changes in conditions [46,47]. Effective nanomaterials such as photoelectric cells, piezoelectric, thermoelectric, and triboelectric materials must be explored thoroughly to meet energy requirements for electroceuticals [46,47].

The device physics with multi-material implementation concerning efficiency and their challenging limitations to match the clinical demands still needs to be explored [48,49]. The fundamental difficulties are safety, reliability, and long-term use that must be addressed to enable potential advantages. All these essential factors come under the root of biocompatibility and material functional performance enabled by functional characteristics such as mechanical, chemical, and electrical properties [48,49]. Devices must effectively extract physiological information by implementing signal processing and machine learning algorithms. At the same time, the device should isolate human biological motions and environmental conditions or detect multiple signals. Considering all the above, developing a miniaturized, lightweight, and battery-free wireless system within an integrated medical device to meet the requirement of portable onsite health monitoring at home or office is a critical challenge.

As we discussed, there are multiple challenges and requirements related to electroceuticals; we hope that the advancement in the field of bioelectronics, with the help of allied areas [50,51], will overcome those difficulties. We believe that these technologies will become feasible from concept to reality in the near future.

## 4. Electroceuticals—Intelligent Environments

### 4.1. Pacemaker

The heart is an electrical system that can regulate the speed and timing of the heartbeat. Heart chambers contract and expand to push blood flow through the blood nozzles. This is how the heart produces rhythms—heartbeat. Sometimes, the heartbeat is not systematic, i.e., in an unsynchronized fashion, and is known as heart failure. Cardio resynchronization therapy is the only chance to eliminate heart failure, and sometimes it may not. A smart biomedical implant, i.e., the biventricular pacemaker, is a device that is implanted under the skin, with two or three wires passing through the veins leading to the heart muscle [52,53]. The biventricular pacemaker has wires in the heart’s left and right lower chambers and tunes the impulses to make the heart muscle contract better and improve the overall pumping function. Suppose the heart goes into cardiac arrest and is in a dangerous rhythm. In that case, the implantable cardioverter-defibrillator produces impulsive currents to the heart muscle to return to a normal heart function. The biventricular pacemaker and implantable cardioverter-defibrillator functions are integrated into a single device that is implanted beneath the skin in the chest. This device has shown a reduced risk of patients with cardiac arrest in heart failure. However, generating impulse-based shocks from time to time under cardiac arrest is most important. If not so, the patient may face serious risks. There are limitations in existing pacemaker devices’ energy supply systems, such as miniaturizing energy storage devices, the battery should supply energy in time, a longer period, etc. The energy storage systems such as lithium-ion batteries efficiently work for a limited time due to their limited charge density and internal resistance. Therefore, the technological innovation in vivo energy harvesters driven by biomedical energy is necessary to supply continuous, uninterrupted power to the titanium packaged implantable devices [54,55,56].

There is ongoing research on bioenergy-driven energy supply systems for heart implants. For example, self-rechargeable cardiac pacemaker systems with triboelectric nanogenerators, thermoelectric energy harvesters, piezoelectric energy harvesters, etc., have been successfully developed [52,54,55,56,57]. Recently, a high-performance inertia-driven triboelectric nanogenerator based on body motion and gravity with the size of a commercial coin has been introduced [57]. Ryu et al. demonstrated the nanogenerator integrated cardiac pacemaker system and conformed self-rechargeable cardiac pacemaker system’s ventricle pacing and sensing operation mode. Self-powered biomedical implants based on piezoelectric harvesters are among the potential candidates that allow and generate electricity from cardiac motion. In this context, researchers have started with lead-based ceramic piezoelectric generators. However, lead-based systems are toxic and susceptible to cracks. Alternatively, polyvinylidene fluoride trichloroethylene-based films were produced as cardiac energy harvesters. Later on, biocompatible and flexible piezoelectric polymer-based nanogenerators were realized as a battery-free heart pacemaker system that could generate electricity from the cardiac motion of the left ventricle [58]. These piezoelectric polymer-based nanogenerators are produced from polyvinylidene fluoride composite nanofibers, zinc oxide, and reduced graphene oxide hybrid nanofillers. The results revealed that these composite fibrous-based nanogenerators produce energy of about 487 μJ. They suggested that this is a viable candidate for implantable applications, and energy is sufficient for pacing the human heart and leads [58].

The developed bioenergy-driven energy supply devices have possessed several potential advantages, such as extending the implantable pacemaker device’s operation time inside the body and reducing the high risk of repeated surgery. However, the implantation of advanced bioenergy used energy supplying elements such as energy harvesters also suffer under implantable environment conditions causing reduced efficiency over time. It may not produce the energy in time for the heart implants. In addition to the above drawbacks, traditional implantable pacemaker devices’ supply leads present the primary threat in most implants. The wireless power transmission technology could be a potential strategy to overcome the above drawbacks associated with traditional developments. This technology greatly eliminates the use of implantable device supply leads [35,37,38,39,59]. Recently, Abiri et al. developed a transmitter-centered control system for wireless power transfer with sufficient power to cardiac pacing [60]. Under the device safety conditions, the device was validated using a computational model. The model results revealed that the wireless system could operate efficiently and exhibit superior tissue energy absorption safety criteria; the quantified value was 50 times lower than the safety conditions [60]. The wireless transfer system based on the magnetic coupling of the magnetic coil was successfully developed by Park et al., which showed 45.5% coil-to-coil coupling efficiency with a coil size 14 mm ×17 mm [35,59]. The experiment results revealed the inductive power transfer link is the most viable for cardiac pacemakers’ and electrocardiogram’ long-term powering [35,59,60]. However, designing an inductive power transmission system without a specific absorption rate for modern leadless cardiac pacemakers remains challenging. The currently developed inductors’ wireless power transfer efficiency is very low, probably attributable to their dimension and implantation depth. The core geometry needs to be optimized to enhance the coupling coefficient, subsequently increase its efficiency. Maybe more optimization studies with the help of simulation tools that could provide field distributions analysis help us increase the inductor’s capacity further. Introducing magnetic core material reduces the temperature increase in the implant area [61]. High permeability materials for the core, even more, the laminated core structures could be potential candidates to overcome the traditional issues and limitations. Moreover, high magnetic conductivity, biocompatibility, low loss property, and flexibility allow us the usage of these materials to develop efficient wireless power transmission systems for heart assist devices such as ventricular assist devices, etc., [61].

The inductive power link employed in the conventional links that must be designed at a high frequency is based on the size, shape, weight, and implantation depth of cardiac pacemakers. Mohanarangam et al. demonstrated a receiver coil with the dimensions 6 mm in diameter and 6.5 mm in length at 13.56 MHz to provide uninterrupted power to the modern leadless cardiac pacemakers [62]. The designed inductive coil dimensions were considered according to the food and drug administration approved regulations of implanted leadless devices. The performance of the designed one was verified through simulations and experimental measurements under perfect alignment, lateral and or angular alignments, and distance variation between the coils [62]. The measured transmission coefficient of the developed coil was about 30.9 dB for a 50 mm horizontal distance between transmitter and receiver coils. The maximum simulated average specific absorption rate at heterogeneous phantom was 0.30 w/kg, which is lower than the limit set by the federal communications commission for radiation threshold exposure [62]. This efficient inductively coupled wireless power transmission system was designed by considering safety regulations and could suggest that the device based on inducting link is a reliable and potential candidate for pacemakers. Recent reports suggest integrating inductively coupled wireless power transmission devices with energy storage devices that could enhance the efficiency towards avoiding abnormal conditions associated with an interruption in the continuous and constant supply of energy in heart implants. However, traditional energy storage elements have limitations concerning the usage of those in biological environments. Furthermore, current energy storage elements face balancing factors, including high-performance, biocompatibility, conformal adhesion, and mechanical compatibility with soft tissues. Hydrogel-based energy storage elements could be potential and viable candidates. Liu et al. successfully demonstrated stretchable hydrogel-based supercapacitors as a potential energy supply source that can supply enough energy to the implants. This hydrogel supercapacitor comprises polyaniline-reduced graphene oxide/Mxenes gel electrodes and hydrogel electrolytes. Its interfaces can have the capability to cross-link robustly, leading to efficient and stable chemical performance. The demonstration results revealed that the device exhibits a high area capacitance of about 45.62 F/g and an energy density of about $333\;\mu \mathrm{wh}/{\mathrm{cm}}^{2},$ 4.68 wh/kg [63]. The potential advantages of these kinds of devices include lightweight, thin stretchable, and wet adhesive with a high area capacitance and ultra-high energy density. This novel device could be useful as a potential candidate to integrate inductively coupled wireless power transmission devices for enhancing the efficiency towards avoiding interruption in the continuous and constant supply of energy could be a novel solution for the heart implants.

Developments in device design, technology, and material science have been established; for example, biological implants realized with the help of 3D printing, bioelectronics fabricated using lithography techniques. Furthermore, bioelectronic sensors with wireless power transmission technology, the internet of things, etc., have been successfully introduced [35,37,38,39,64,65,66]. Electronic sensors based on microelectromechanical, magnetoelectric resonance, and capacitive sensors to detect pressure flow, velocity, etc., have also been successfully developed [67]. However, many limitations still oppose using these existing technologies implementing and integrating them as multifunctional electronic components in cardiovascular applications. Material selection is a crucial and most essential factor for implementing and integrating them in cardiovascular applications. The integrated devices and the materials must be biocompatible and should not induce any undesirable effects on the surrounding biological tissues. It should also have the ability to assist or heal the disease or record signals after implantation while providing a stable connection between the external device and the implant surface. Importantly, integrated devices and materials must be tested for toxicity, carcinogenicity, hermeticity, and material degradation over time to achieve efficient performance towards heart functions under safety regulations. With the help of allied areas and technologies [50,51], we hope that this advancement will soon become feasible from concept to reality.

### 4.2. Neural Stimulations and Electrode Arrays

**Figure 2:**
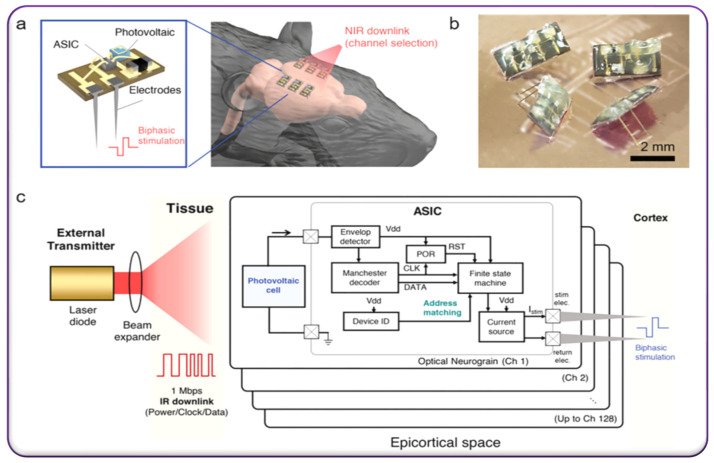
Wireless deep brain neural stimulator (**a**) device model and implanted device in a rat model illustration, (**b**) illustration of 4 fabricated implants, (**c**) overall concept block diagram and its electronic configuration consisting of external near-infrared (NIR) source transmitter for providing an optical wireless link to the downlink to stimulate optical neurogranin. Reprinted with permission from Lee et al., ACS sensors 6, no. 7 (2021): 2728–2737. Copyright 2021 American Chemical Society.

The brain plays a significant role in all functions and activities of life, controlling cell growth, biological and neural activities, physical motions, and many more. Hence, understanding and controlling brain functions have always been an ambitious goal for researchers and industries (Table 1). A bioelectronic device such as the deep brain stimulator has successfully helped patients in treating Parkinson’s disease or related neurological disorders, and recently, further technological enhancements such as current directing electrode configurations, neuromorphic electronics, synaptic devices, brain chips, etc., have been pursued for the next generation’s stimulation system [68,69]. Lee et al. successfully developed an implantable device to activate brain neurons. It consists of three parts, a small module of extracranial brain activator, a base station for transmitting power wirelessly, and an intelligent device for monitoring and controlling operations [70]. A small module of a coin size was implanted under the brain’s scalp, the base station transfers the power wirelessly to the implanted device to stimulate neurons, and the intelligent device monitors and controls all operations. The pre- and post-stimulation studies revealed noticeable brain activity after injecting stimulative signals. Further, this suggests that brain activity causes a significant improvement in memory and sensory skills [70]. Alivisatos et al. have focused on the operational effectiveness of brain implants for obsessive-compulsive disorder epilepsy and Parkinson’s disease [71]. Moreover, the research group extended their attention to other areas such as chronic pain, traumatic brain injury, depression, stroke recovery, etc., [71]. However, developing a low-power wirelessly compatible brain-stimulating device is a significant challenge due to their operational and device compatibility limitation in real-time. Lee et al. have successfully introduced a miniaturized implantable optoelectronic brain stimulator [72]. The device operates with low power and consists of integrating application-specific integrated circuit (ASIC) and triple junction gallium arsenide (GaAs) based photovoltaic (PV) unit fabricated via microfabrication technology to stimulate the cortex, which is shown in Figure 2a,b. The design feasibility was demonstrated on the benchtop and in vivo acute rat model [72]. The developed cortex stimulator and its electronic components assembly representation are illustrated in Figure 2. The utilization of infrared energy as an energy source supplying wirelessly to an implanted device to generate sufficient stimulative responses is mainly because of their larger wavelength nature that can easily and deeply penetrate the human biological material without loss of energy. Furthermore, the infrared wave wavelengths match the organic and inorganic human body material wavelengths, meaning organic and inorganic biological material’s resonance frequency is in the range of infrared wave frequency. Therefore, the infrared waves could deeply penetrate the body and supply sufficient energy to a PV cell without creating any abnormal conditions for the biological cells. In this integrated deep brain stimulator system, the gallium arsenide (GaAs) based photovoltaic (PV) device efficiently converts the incoming infrared energy to electricity [72]. The chip of Manchester downlink efficiently delivers the converted incident power to the electrodes to trigger stimuli by remote command (Figure 2c). The developed integrated assembly of the photonic-electronic chip within a small area of 2.9 mm × 1.7 mm having wireless capability helps address all relevant issues during stimulation [72]. If the device-defined coding sequence matches the incoming downlink bit sequence among the multiple devices on neurogranin, the device activates the stimulations. The implant delivers the electric charge that is converted photons to electrons to the targeted cortex [72]. This kind of advanced development with advanced circuitry enabled by advanced nanofabrication technologies would be a potential candidate for deep brain stimulations. Furthermore, advanced nanofabrication technologies would allow us to design deep brain-stimulating devices based on a large number of electrode nodes for applying space-time patterned microstimulation across multiple cortex nodes. These devices would be an alternative to traditional wired, high-power, and bulky devices.

In addition to deep brain stimulations, low-power neural signal recording devices are essential for understanding the physics behind neuron dynamics. Based on which advanced brain-stimulating devices with novel concepts would come out, also it can help the clinician treat the patient better. However, low-voltage-based neuron signal recording electronics exhibit noise. It generates an abnormal noise while recording the signals during deep brain stimulations or from naturally generated neural signals and neuronal dynamics. In some cases, this noise may cause disturbance or inconvenience to the patient. An integrated low drop-out (LDO) voltage regulator can overcome this drawback. LDO voltage regulator detaches the supply of the analog front-end from the output of a rectifier or a noisy DC–DC converter. However, low dropout voltage instigated loss of power-effectiveness would increase the energy required to the device to their operation. Low drop-out voltage powered device efficiency can be calculated with the following mathematical expression [73].

$$\eta_{\mathrm{LDO}}=\;\mathrm{Vout}/\left[\mathrm{Vout}+\mathrm{Vdo}\right]$$

In which η is the efficiency of LDO voltage regulator: Vout−output voltage and Vdo−dropout voltage. Let us assume a ~97% efficiency of the DC–DC converter with a 110 mV dropout-voltage of an LDO voltage regulator powered the analog front-end device. The entire system exhibits a power efficiency of <80% when the output voltage is below 0.5 V. This low efficiency is attributable to the power loss of the LDO voltage regulator. Although reduced drop-out voltage could improve the system efficiency while reducing noise, it necessitates a large-sized power transistor, causing the design of an LDO voltage-regulator with a high power-supply-rejection-ratio, which is further challenging. An analog front-end device’s high PSRR (power supply rejection ratio) would be the proper choice to circumvent the above difficulty. It eliminates the requirement of an inefficient low-dropout voltage regulator, reducing the required power while minimizing the noise. Thus, one of the research teams implemented the strategy in developing a wireless brain neural signal recording system with ultra-low power utilization capability, illustrated in Figure 1 of [73]. Lyu et al. demonstrated significant efficiency of developed deep brain signal recording system. The designed device consists of an 8-channel energy-efficient analog front-end with high PSRR. A replica biasing scheme was used to maintain high PSRR while maintaining low supply voltage [73]. The input stage in the low noise amplifier adopts a low supply voltage of about 0.35 V, where the current is re-using to achieve ultra-low power. Finally, they suggested that this device concept utilizing advanced functional materials would facilitate the development of future low supply voltage-based neural signal recording devices and deep brain-stimulating devices [73]. In addition to requiring a low power supply with miniaturization capability to the deep brain stimulating and signal recording electronics, efficient wireless power, and data transmission capability is also desirable. Though subcutaneous stimulations are promising over transcutaneous stimulations [74], to enhance neuromodulatory effects in the brain, De Marcellis et al. have introduced efficient transcutaneous telemetry design with an enabled machine interface [75]. The subsystem uses a nanosecond laser pulse to achieve a high data transfer capability with relatively low power levels [75]. This is a significant advancement in brain devices compared to the other wireless power and data transmission systems and methods. The system has developed with the help of components such as semiconductor laser and driver, fast response Silicon photodiode, interfaces integrated on a single board, and reconfigurable logic device FPGA-based encoder-decoder processing circuits. The demonstrated results reveal that the system can be capable of operating a data rate unto 300 mbps with a bit error rate bar of less than $10^{-10}$ and energy efficiency of 37 pJ/bite. The system has the ability to communicate 1024 channels of board neural data sample at 18 kHz with only 11 mw power consumption [75]. However, a significant challenge is to develop an efficient signal modulating algorithm for wireless transmission networks to analyze and collect biological data while avoiding intra-personal variability effects induced signal interference. Park et al. proposed a novel method called tissue property-based approach: direct parameterization from the physical properties of a layered tissue body structure for quantifying intra-personal variable effects [76]. This approach could be useful for developing an equivalent circuit model-based algorithm that integrates with the signal modulating technique to collect and analyze the responses of tissues to the stimulations without variability-induced effects.

**Figure 3:**
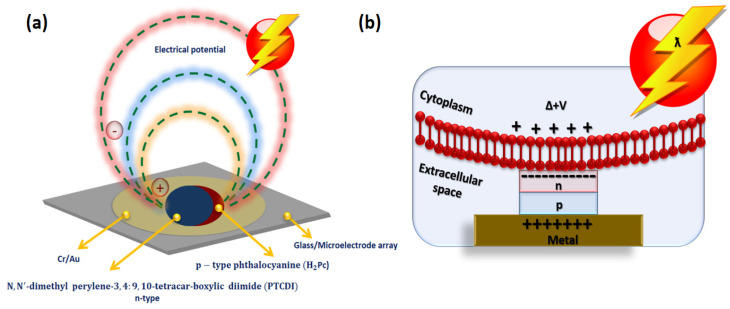
Representation of photo-capacitor device (**a**), and capacitive coupling mechanism of photo-capacitor device (**b**) [78].

Biophotonics such as implantable photonic probes, etc., have gained interest in optogenetic neural stimulation [77]. However, these photonic probes can create excessive heating to the tissues. The light diodes can produce more heat than light; the resultant temperature rise from the probe surface needs to be maintained under a regulatory limit of 2 degrees. This can be optimized by combining optical and thermal modelling methods. Analysis of the optimized design may help fabricate more effective probe emitter geometries with adequate illumination volume under thermal limitations [77]. More recently, direct electrostimulation via a semiconducting system was reported by Rand et al. [78]. This electrolytic-photo-capacitor device consists of 80 nm thin metal layer and p−n semiconducting pigment layer of 60 nm, device architecture is shown in Figure 3a. The photo-capacitor capacitive-coupling mechanism and the adjacent cell interactions are illustrated in Figure 3b.

This device charges up when it is under photo illumination energy. When the physiological solution illuminates, the metal–pigment-based p−n junction device interacts with the living cells and stores the electric charge with the capacitive coupling effect. Thus, the transmuting of light-wave energy into electrical energy. Electrical energy is in the form of electrostatic currents that are adequate to excite neurons. This electrolytic-photo-capacitor device has potential advantages such as no external bias and no wiring requirement and is consistent under biological circumstances. The device uses a biologically compatible and non-toxic semiconducting pigment layer enabled by established deposition techniques [78]. On this photo-capacitor film, primary neurons were cultured, then the efficiency of the device was demonstrated by direct optoelectronic stimulating light insensitive stimulation neurons, providing a potentiality of this device for neural stimulations. The device technology could be a thought for monitoring generated false frequency signals by comparing stimulative impulsive, direct current signals from the capacitor over an area. This powerful technology can also be a useful path for developing novel neuron stimulating devices.

**Figure 4:**
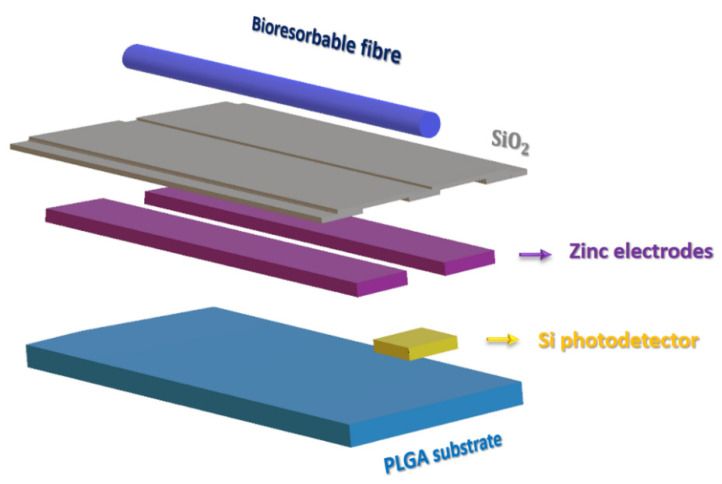
Pictorial representation of the device, its assembly [79].

Additionally, a neural stimulating device’s real-time internal physiological processes monitoring capability could facilitate the information required for generating stimulations as a part of surgical intervention procedures [79]. Existing approaches depend on imaging technologies or sensors that are implantable. These are unsuitable for providing unremitting data statistics over clinically relevant time scales, also requiring surgical procedures in addition to the associated costs and risks. Alternatively, Bai et al. introduced the injectable photonic device, device assembly representation shown in Figure 4. The device was prepared by the functional material that can resorb, and after a specific time, it undergoes clearance from the body to characterizing targeted tissues and biofuels. The injectable photonic system consists of bioresorbable optical components, including nanomembranes based single-junction photodetector, tricolor stacks of silicon p-n junction based tricolor photodetector, and optical multilayer filters of ${\mathrm{SiO}}_{X}$ and ${\mathrm{SiN}}_{Y}$ optical-fibers of poly (lactic-co-glycolic acid). Demonstration of this system includes the device that injects into a deep brain to monitor unceasing biochemical absorption and physiological parameter analysis. The results of physiological studies revealed monitoring of cerebral oxygenation and neural activity that suggests the device’s accuracy and viability. Analysis of biodistribution, blood count, and blood-chemistry highlight the process by which these systems undergo bioresorption [79]. Clinical results showed the device that could continuously monitor oxygenation status, cerebral temperatures, and the action of the neural system in mice [79].

The device technology would also be helpful in monitoring and assessing the parameters relevant to tissue metabolic-process and tissue health [80]. Assessment of the metabolic and physiological pathways associated with acute diseases is essential and would be useful for initiating specific, timely, and effective therapeutic stimulations to a neural system [81,82,83]. Measuring local changes can serve as a basis for delivering the scheduled optimized impulse signals as a part of the recovery rehabilitation protocol [84,85,86]. Further, these biological functionalities assessing technologies can be useful for understanding fundamental concepts of diseases pathology, surgical procedures, and monitoring recovery from injury or illness.

The fundamental study of the brain, how it works, its operations, and signal recoding is the most important and major challenge of neuroscience. To do this, implantable multielectrode array-based devices are promising tools. Cellular spatial resolution, sub-milli-second-time precision, both spiking and low-frequency bioelectric signals from the electrode array can help diagnose the patients’ disease functional characteristics more efficiently [87,88]. Integrated implantable single shaft probes with dense electrodes and probe circuits were developed [89,90,91]. Implantable deep brain-stimulating electrodes via complementary metal-oxide-semiconductor technology have been successfully fabricated [89,90,91]. The current advancement in semiconductor technology can help overcome routing electrode pad interconnections that typically limit the electrode density and number of electrodes in micro nanostructured passive probes. Further, this approach helps integrate chip front-end circuits with high fidelity and high-density microelectrodes for extracellular recording [92,93]. Recently, Angotzi et al. successfully developed active pixel sensor probe technology via semiconductor fabrication technology for simultaneous neural recording. The recorded acute neural bioelectrical signals from each electrode successfully resolved and discriminated the activity from several packed neurons both at a special and temporal scale [94]. The team showed the reliability of this device. However, the team has not demonstrated the device is helpful in a convenient way in vivo due to its complex nature. Contactless technologies are desirable to avoid the complexity associated with the device for determining biological tissue electrical properties with high sensitivity, usually employed by a radio frequency or microwave technology. For example, miniaturized high-temperature superconducting coils, multiturn split conductor transmission line resonators, or flexible polymer-based antennas have been demonstrated recently as contactless technologies for brain electronics [95,96,97]. Recently, a wireless probe has been developed for sensing dielectric properties of tissues, which is noninvasive, noncontact, and easy to implement diagnosis of tissues in vivo [98]. This sensing technology involves distance monitoring with a high-quality factor radiofrequency resonator that is electromagnetically coupled with the investigated tissue. The preliminary sensing results showed the effective electrical conductivity and the permittivity of considered phantoms [98]. These tissue characterization and neural recording probe technologies open up novel ways to develop new devices for further understanding the biophysics behind those. This significant information is essential to deliver the desired stimulations in time to a specific disorder.

CMOS chips are necessary for collecting, processing, and transmitting the data for all the above developments [99]. Postprocessing miniaturized CMOS chips by usual methods are challenging. Typically, sub-mm size components are hard to handle and not readily consistent with standard microfabrication methodologies such as photolithography, etc. Recently, Lee et al. proposed a soft material-based scalable post CMOS processing method that enables photolithography and electron beam deposition to produce hundreds of micrometers-scale components [99]. They successfully fabricated electrophysiological recording and a microstimulation device to assess functional electrical properties. The device consists of monolithic integration of patterned gold and PDOT: PSS electrodes produced within a small microscale area and was used to evaluate their functional electrical properties [99]. The outstanding response recording and stimulation of microscale interfaced electrodes of the developed device were verified in saline and in vivo experiments using wireless power and data links [99]. Thus, the report suggests precisely aligned CMOS chips can be realized by enabling batch postprocessing without complication from the additional micromachining or chips treatments. Moreover, a report suggests advancement in the semiconducting industry is essential to develop more complex and functional neural stimulation devices, and their electrode systems help to monitor and treat abnormal neurological dynamics. That could be beyond CMOS refer to the digital logic technologies that suppress the current CMOS scaling limits and more. With the help of beyond CMOS technologies, researchers hope to meet the demand for either electrically or magnetically accessible memories that could enable high speed, high-density data storage component design and their embeddability. It is essential for the neural stimulation device industry.

The semiconductor industry appears to be reaching the limits of miniaturization with limited technologies and material resources. Therefore, researchers are gaining attention in developing new materials that compete with the existing material of CMOS technology. 2D Graphene and related 2D materials have great potential to overcome these limitations, and that could be more viable with the beyond CMOS technologies. Moreover, compound semiconductors are also a part of beyond CMOS, other than silicon, such as gallium nitride and gallium arsenide, etc. These compound materials can operate at higher frequencies and emit and detect light more efficiently. Hence, there is great potential for wireless energy transmission technologies such as Wi-Fi to neural stimulation devices. Li-Fi, i.e., light communication, is another future technology for communicating recorded data and supplying energy to neural stimulation devices. We believe that beyond CMOS technologies that could enable utilization of compound semiconductors and other component integrated circuits and facilitate in developing novel functional neural stimulation devices and their members for telemetry-based neural stimulation devices. High-speed communication is also viable with these technologies.

### 4.3. Artificial Retina

Nearly 40 million people have blindness worldwide, and another 124 million people are under the low vision category [100,101,102,103,104,105]. Researchers are trying to find solutions to solve vision problems. There are two main approaches to improve vision. The first one is retinal prosthesis: within this, two methods are available: epi-retinal stimulation and subretinal stimulation, which involves implanting an electrode array between bipolar cells and retinal pigment epithelium. Compared to epi-retinal implantation, the subretinal prosthesis has an advantage such as closer proximity to the neurons to stimulate and helps in improving visual perception. The second one is the optic nerve, about 1–2 mm in diameter, which serves as a compact conduit for all information in the visual sense. This suggests that stimulating the optic nerve even in a small area would cause greater improvement in the visual sense. This approach offers a safer and easier implementation procedure compared to other techniques. However, focal stimulation and detailed visual perception are difficult due to the stimulating electrodes located over the densely packed optic nerve [100,101,102,103,104,105]. Developed technologies by the companies in Table 1, on both the approaches for restoring vision have yielded the best outcomes, authenticated by the implemented clinical trial experiments. Some of the clinical studies (some of them are ongoing) of these implanted devices both near the optic nerve and retina have revealed that they are safe and can be implantable [100,101,102,103,104,105]. Although the reported clinical experiments show the efficiency, safety, and compatibility of those, vision with a relatively low spatial resolution of developed implants confirms that further device development is still needed to achieve improved vision and functional vision [100,101,102,103,104,105].

**Figure 5:**
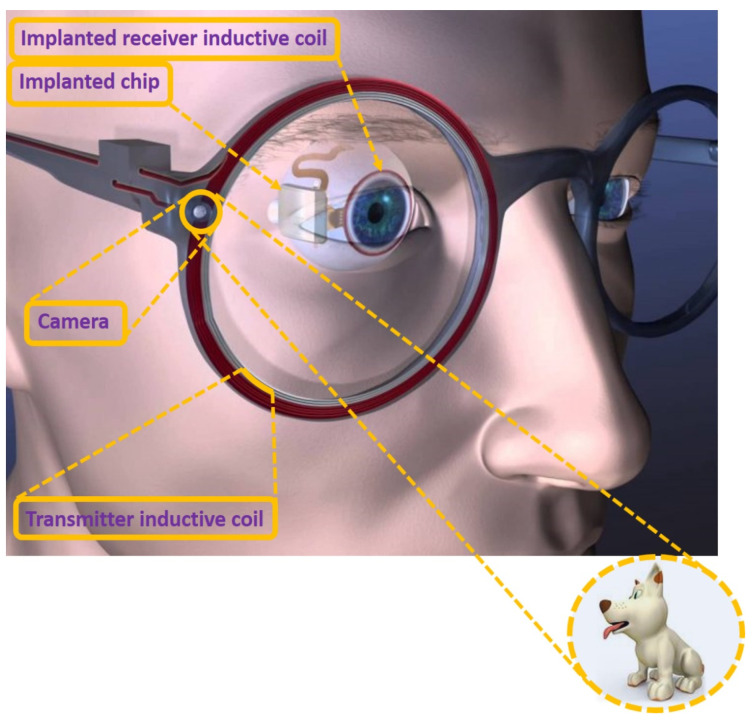
Boston bionic implant: concept of Boston bionic implant [106,107,108].

One such development is the bionic eye; Boston artificial retina, illustrated in Figure 5 [106,107,108]. The device consists of an external transceiver and implanted hermetic neural stimulator. When the patient wears eyeglasses, the camera mounted on the eyeglasses captures an image and sends it to the intelligent controller unit. The controller unit process and encode the image data. Then the encoded data is sent to the implanted device wirelessly, where implanted device antennae receive the encoded data. Then the processing system of the implant decodes the data and induces impulsive electrical signals to stimulate retinal tissue to create artificial vision [106,107,108]. Here, the process of sending power wirelessly to the implant requires a compatible and biologically safe method. State-of-the-art research suggests that inductive coupling is the potential and significant source for transmitting power to the implant by assigning one inductive coil as transmitter antennae and the other one as receiver antennae among the two inductively coupled coils [35,37,38,39]. Additionally, a sophisticated electronic circuit is desirable to process, control, and trigger stimulations’ exact size and shape. Furthermore, charge-efficient and biocompatible metallic electrodes are required to safely transport stimulating currents to the earmarked tissues. The fabricated electrodes must be compatible in micro/nanosize configurations or planar or semi-curved shapes and should be biologically corrosion resistive and hermetically sealed. The entire electrode system must be capable of working within the intelligent biological environment. An advanced electrode array, mainly in large numbers, is desirable to trigger in a wide area under associated safety conditions. Furthermore, all the advanced circuits with digital control features integrated on a single chip within a small area mean it miniaturized and compatible single-chip design is the most important consideration for the retinal implants along with the device that works under safety regulations [106,107,108]. With all the above desirable considerations, the Boston team has developed the implantable artificial retina; the retinal implant concept is shown in illustrated Figure 5 [106,107,108]. The retinal implant components are located outside the eye and are attached to the eye’s outermost layer, the sclera. The implanted electronic chip is placed behind the conjunctiva, the thin membrane attached to the eyelids that overlay the sclera. The electrode array is inserted in the sub-retinal space, between the retina and the choroid, delivering stimulus current to the retinal nerve. To sustain the device under intelligent, intellectual, biological atmospheric conditions, the device must be isolated by a biologically compatible and electromagnetic field-compatible sophisticated functional material. Therefore, the Boston team used metallic isolation over the telemetry system chip, a safe isolation material against electromagnetic interference, and is hermetic [106,107,108]. Although metallic shielding is immune to electromagnetic interference, researchers and biomedical engineers are thinking bioceramics utilization, probably due to an advanced hermeticity. However, room temperature processability and frequency respective functional properties limit their usage. We hope the advancement in the bioceramics field will help use bioceramics in the packaging of biomedical implants soon.

Initially, the Boston team introduced a first-generation retinal implant, shown in Figure 2 of [108]. Later, they introduced a second-generation device with miniaturized, integrated, and more advanced electronic components in a compact design under safety regulations to increase the resolution of artificial vision. The second-generation device concept is illustrated in Figure 1 of [106]. The new second-generation design overcame the three limitations outlined earlier with the first-generation device. The first one is the coil design, which is designed in such a way that the coil surrounds the cornea under the conjunctiva comfortably and efficiently coupled with the transmitted coil antennae. The second one is the hermetic metallic sealing of the entire bionic chip, excluding the wirelessly connected coil and the electrodes. The third one is an advanced superior electrode array design to provide efficient stimulations into subretinal space [106,107,108].

**Figure 6:**
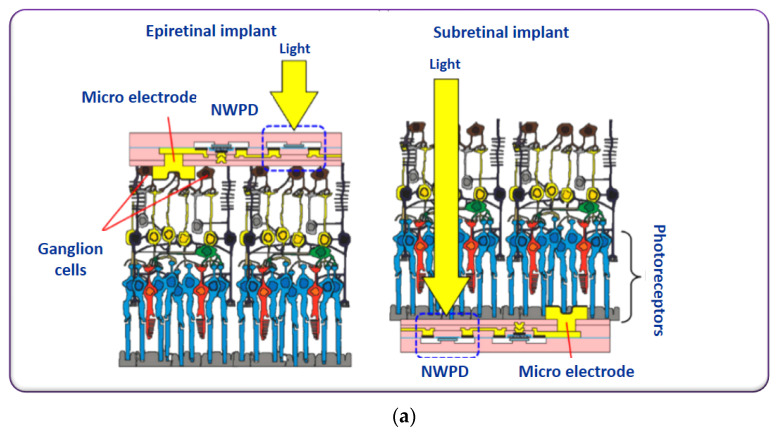

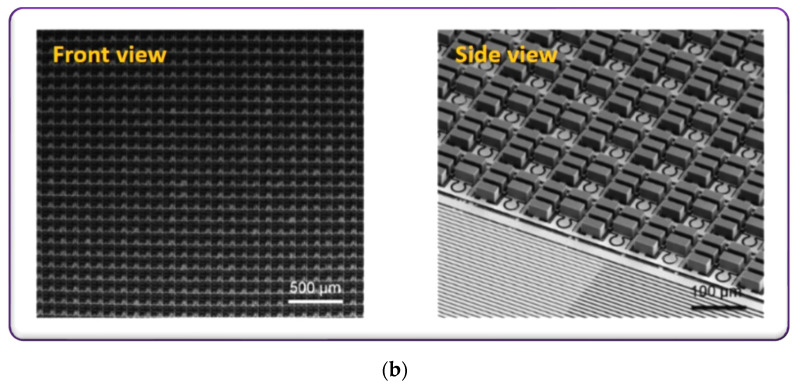
(**a**). The concept of the neural stimulation device is to activate neurons with light energy. Reprinted with permission from Suk Won Jung et al., Sensors 16, no. 12 (2016): 2035. Copyright 2016 MDPI Publishers. (**b**) Scanning electron microscopy images: fabricated for neural stimulation device. Reprinted with permission from Suk Won Jung et al., Sensors 16, no. 12 (2016): 2035. Copyright 2016 MDPI Publishers.

**Figure 7:**
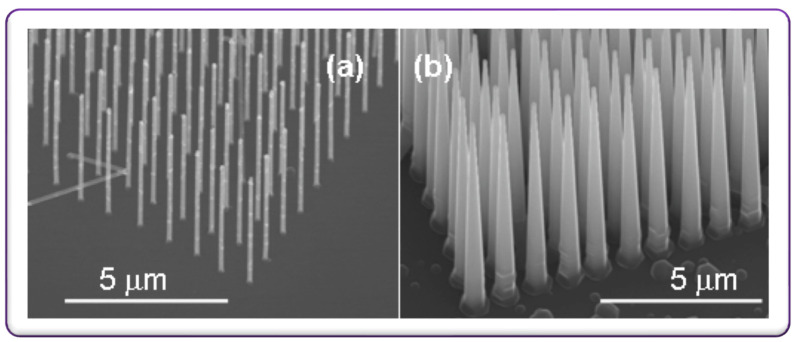
FESEM images of fabricated Si nanowire arrays (**a**) rod shape (**b**) cone shape at different molar ratios of ${\mathrm{SiCl}}_{4}/H_{2}$ using e-beam lithography may be helpful for neural stimulation. Reprinted with permission from Krylyuk et al., ACS nano 5, no. 1 (2011): 656–664. Copyright 2011 American Chemical Society.

Diseases of the retina, for example, retinitis−pigmentosa: abnormality in photoreceptors function are the leading cause of blindness in most cases. To restore vision, alternative approaches are stimulating the photoreceptors’ function or replacing the function of the photoreceptors [109,110,111]. Recently, Akinin et al. demonstrated an on-chip inductive coil-based power transfer system for electrically controlled, optically addressed retinal prosthesis [112]. The device consists of a group of vertical Silicon nanowire photodiodes connected by a transparent conductor and topped by a sputtered iridian oxide electrode. Light is the energy source to induce current from the nanowire-microelectrode-array and is controlled by the inductively coupled power. The electrode-based system is implanted in sub-retinal space since currents as an output of each electrode are proportional to both incident light energy and applied voltage. This is how the system generates the specific shape/timing of biphasic stimulating current to the neurons to fire. The entire electrode system is also feasible for producing a range of stimulating currents depending on the wavelengths of incident light [112]. The optical energy-based addressing retinal prosthesis with electrically activated electrode array operating principle is depicted in Figure 3 of [112]. The Jung et al. group reported a similar implantable retinal prosthesis device with an integrated silicon nanowire-based photodetection circuit to activate the retinal neurons with light energy [113]. This proposed device has the capability to be implemented in both eyeball sites, epiretinal and subretinal sites, known as epiretinal implants and subretinal implants, as visualized in Figure 6b. In epiretinal implants, the electrodes are in contact with ganglion cells. The light is incident on the opposite side where the microelectrodes are formed and reach the nanowire photodetection circuits (NWPDs). While in the case of subretinal implants, microelectrodes are contacted with photoreceptors. The light is incident on the same side where the microelectrodes are formed and reaches the NWPDs. Scanning electron microscopy images of the fabricated device are illustrated in Figure 6b. The prosthesis device consists of a voltage regulator and current driver in which silicon nanowires help as similar functions of a photodetector and field-effect transistor such as detecting light and generating stimulation signals according to the light intensity [113]. The device demonstrations suggested that this generated stimulation pulses to retinal tissues; the ratio is high and directly proportional to the light intensity. Therefore, this significant development is a step forward in retinal prosthetic systems that has the enhancing visual resolution capability. Recently demonstrated silicon nanowire arrays shown in Figure 7a rod shape Figure 7b cone shape at different molar ratios of ${\mathrm{SiCl}}_{4}/H_{2}$ fabricated via e-beam lithography, could be thought of as a novel electrode system for retinal prosthetic devices [114] because of their systematic periodic arrangements and can be integrated with the retinal implant to deliver the stimulative signals to the retinal neurons at a constant field intensity and currents over a wide band of light energy while maintaining uniform stimulative signal distributions. With this significant advantage of the electrode array system, a high-resolution retinal prosthetic system that competes with traditional ones could be realized.

**Figure 8:**
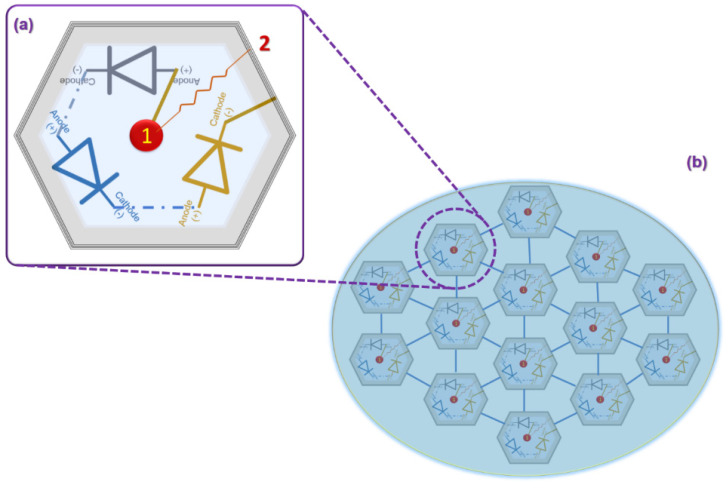
(**a**) The electronic circuit configuration of a single photovoltaic electrode, (**b**) Interconnected photovoltaic arrays electrode system for subretinal stimulation [115].

Lorach et al. developed novel subretinal implants based on photovoltaic arrays, which induce localized stimulations to the retinal neurons revealed after experimenting on rats [115]. The photovoltaic array module and its electronic configuration are illustrated in Figure 8a,b. The photovoltaic module comprises a 70 µm wide photovoltaic element separated by 5 µm trenches arranged in a 1 mm wide hexagonal pattern. The circuit configuration of each photovoltaic element is shown in the subsequent Figure 8a. Each photovoltaic window consists of three photodiodes connected between central active (1) and surrounding return. The visible light triggers the stimulations through the photodiodes located between central active and surrounding return. At the same time, much brighter pulsed illumination generates the biphasic pulses of current in the stimulating photovoltaic cell [115]. All the above electrode configurations and systems are gaining energy wirelessly. Among them, photo energy-based prosthetic systems with high contrast, spatial resolution, and miniaturized capability could greatly stimulate the photoreceptors’ function or replace the function of the photoreceptors for restoring vision in visually blinded persons. This significant advantage is because of their adaptability within the retinal system and operation capability by gaining energy from the light energy. However, these devices require a battery system to store energy, which can be delivered as stimulative responses when photovoltaic energy is unavailable.

**Figure 9:**
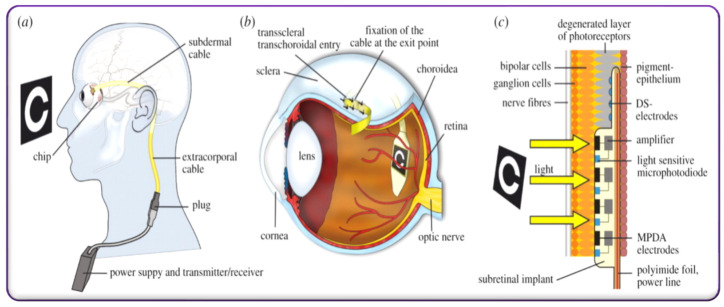

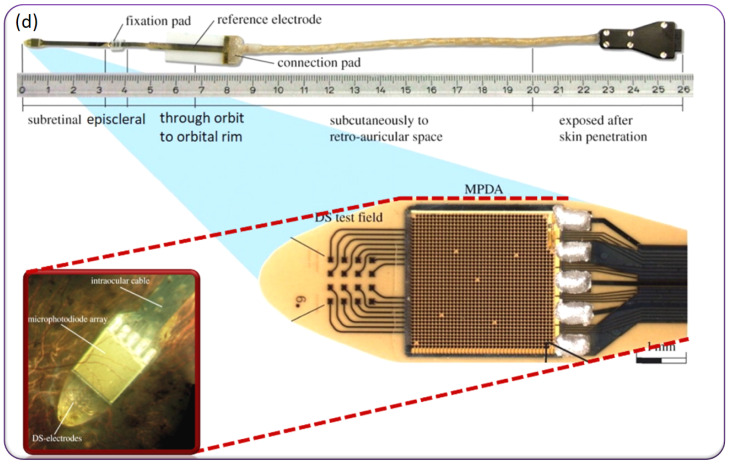
(**a**) The cable connection and end-to-end (from retinal implant to outside transmitter/receiver) assembly of implant system, (**b**) position of an implant under transparent retina, (**c**) illustration of stimulating system concept; photodiode, amplifier, and electrodes. Reprinted with permission from Zrenner et al., Proc. R. Soc. B (2011) 278, 1489–1497. Copyright 2011 The Royal Society. (**d**). Illustrates the entire device, electrode system, and photograph of the subretinal implant tip at the patient eye. Reprinted with permission from Zrenner et al., Proc. R. Soc. B (2011) 278, 1489–1497. Copyright 2011 The Royal Society.

Zrenner et al. demonstrated an implantable microelectronic chip concept, i.e., a micro photodiode array implanted in sub-retina space near the macular region [116]. The retina with the embedded chip is an alternative way of restoring the function of the photoreceptors. The implantable device principle concept is illustrated in Figure 9a–c. The chip consists of the electrical circuit, photodetector, amplifier, and stimulator (Figure 9c). Additionally, light-independent electrodes were introduced at the device’s tip to produce direct stimulations and test connectivity. An array of 1500 active micro-photodiodes integrated with its own amplifier and stimulating electrode. The light activates the photodiode, and the connection electrodes stimulate the subretinal neurons (Figure 9d) [116].

The advantage of this device is that all parts of the device can be implanted invisibly in the body, that inner retina processing can be performed continuously and stably to achieve a stable image with special high resolution [116]. However, further device advancement is needed to establish long-term stability, contrast, special resolution, and miniaturization capability. The current study provides a proof of concept that electronic subretinal prosthetic devices can improve the visual function of visually blind patients. An organic electrolytic photo-capacitor was recently introduced by Rand et al. [78]. The device comprises a thin 80 nm tri-layer of metal and p-n semiconducting organic nanocrystals. This metal-semiconductor device charges up when it is under photo illumination energy. When the light energy illuminates the physiological solution, it transduces light pulses into electrical currents. Electrical energy is in the form of electrostatic currents that are adequate to excite subretinal neurons. The electrolytic-photo-capacitor device has potential advantages such as no external bias and no wiring requirement and is consistent under biological circumstances. The device uses a biologically compatible and non-toxic semiconducting pigment layer enabled by established deposition techniques [78]. The device’s efficiency was evaluated by collecting direct optoelectronic stimulation intensities of subretinal neurons. This device is also useful for stimulating electrogenic tissues [18]. Combining photo-capacitor-inspired energy storage devices with photon energy-based stimulating sources would open up novel solutions to restore vision in patients [115,116]. For example, integrating electrolytic photo-capacitor energy storage devices with photovoltaic array-based wireless restorative electrodes or micro photodiode array-inspired triggering electrodes or optically addressable electrically controllable nanowire microelectrode array-based retinal prosthesis systems could help to restore vision artificially. These significant developments within an integrated atmosphere as a compact design would greatly enhance visual resolution in blended patients attributable to their storing the photo energy, subsequently retinal neuron stimulating capability.

**Figure 10:**
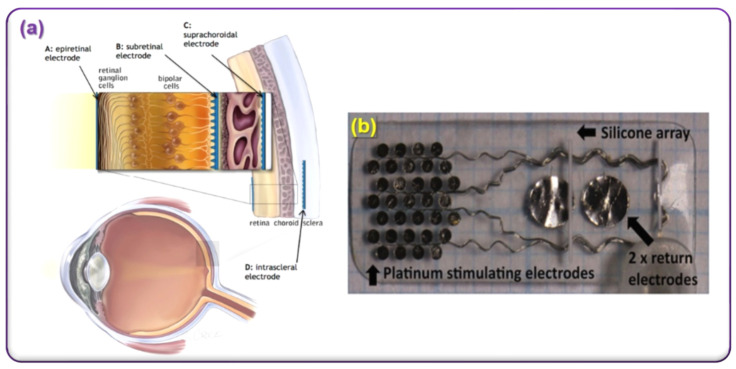
(**a**) The concept of intraocular electrode array-based implantable device, (**b**) Fabricated intraocular electrode array. Reprinted with permission from Ayton LN et al., PLoS ONE 9(12), e115239 (2014). Copyright 2014 PLoS ONE.

First, a human trial, a novel suprachoroidal retinal prosthesis, was conducted by the team of Bionic Vision Australia (Figure 10) [117]. During the experimental study, the device revealed a key advantage, which is reducing the risk of retinal damage. The benefit attributable to the suprachoroidal site is that the electrodes do not contact the neural retina, which is clearly shown in Figure 10a. In addition, the surgical technique does not require integrating the device within the vitreous cavity, negating the need for a corneal incision, intraocular lens extraction, vitrectomy, and retinal surgery [117]. This implant does not disturb natural vision because it does not interact with the eye optic neurons. Unlike epiretinal devices, which block natural vision, developed implantable device output instigated vision could coexist with a natural vision. The materials used in this device are platinum, titanium, and titanium-copper-nickel-alloy (TiCuNi) (hermetic encapsulation material for the first developed pacemakers) and biocompatible silicon-elastomer. The device consists of a medical-grade silicon substrate, over which the platinum electrode array of 19 mm ×8 mm and two large electrodes were developed. The fabricated intraocular electrode array is illustrated in Figure 10b. In this study, a percutaneous connector was used as a direct electrical connection, allowing flexible neurostimulation and electrode monitoring without the need for any implanted devices [117].

Though the number of developments with different electrode configurations revealing reliable retinal prostheses, more recently, the advanced materials-inspired electrode systems are attracting for implantable devices, neural interfaces, and other biomedical fields due to their enhanced electrochemical performance, electrical activity, and biocompatibility [118]. For example, a hydrogel-electrode system was developed on the platinum substrate with the modified poly (3,4-ethylenedioxythiophene) polystyrene sulfonate (PEDOT-PSS) via electrochemical gelation process, employing copper as a sacrificial layer [118]. The morphology helps the hydrogel-based electrode to achieve a significantly low impedance down to $10.09\;Ω{\;\mathrm{cm}}^{2}\;\mathrm{at}\;1\;\mathrm{kHz}$. This conductive polymer hydrogel electrode material provides an extensive large surface area with good adhesion. Such material presents a great potential for preparing flexible, functional electrodes imploying promising applications in neural implants [118]. Combining photo-capacitor-inspired energy storage devices with hydrogel-based electrodes would open up novel solutions to restore vision with high resolution while maintaining biocompatibility in patients [115,116]. Furthermore, integrating a photovoltaic array with hydrogel electrode-inspired stimulating sources or micro photodiode array adopted hydrogel electrode systems would be useful for efficient retinal prosthesis. However, developed devices with clinical authentications must be implemented in real-time applications as implantable devices.

Although the above introduced retinal prosthesis systems and sub-systems, such as the bionic eye, retinal implants, and their electrode systems, etc., are authenticating with clinical experiment results, they do not restore vision/sight to the extent [119]. The limitation is largely due to the current implants that have a limited number of electrodes in single or double digits. Millions of electrodes would be needed to provide high-resolution vision equivalent to that of natural vision with the help of electronic retinal prosthesis systems and subsystems. This might be a complex challenge now. We believe that advancement in fabrication technologies and material science will overcome the above significant challenge in the future. Currently, reports suggest that using brain implants can stimulate vision sensory neurons that could greatly improve vision in patients. Researchers also suggest that brain anatomy could influence the loss or absence of vision. This significant advantage is because one-third of the human brain neuron system is devoted to vision. Therefore, we believe integrating implantable retinal prosthesis systems with vision sensory neuron stimulators in the brain aiming to substitute visual activity and capacity would be a novel solution [119].

### 4.4. Vagus Nerve Stimulation

The vagus nerve consists of a heterogeneous group of neural fibers innervating numerous organs. Vagus nerve stimulator (VNS) broadly stimulates the connected heterogeneous fibers. It is a neuromodulation system that can be implantable and consists of a pulse generator implanted under the skin near the chest [120]. It runs lead wires up to an electrode cuff wrapped around the cervical bundle of the left vagus nerve [120]. Jake Zabara first introduced it in 1980 and experimentally proved antiepileptic effects [121]. Globally, many patients had a vagus nerve stimulator system implanted in their bodies [121] for treating epilepsy, depression, and migraines [122,123,124,125]. It is under investigation for treating pathological conditions such as motor impairment after heart stroke and tinnitus [126]. The VNS system’s first controlled clinical trials were conducted in 1990 to treat refractory epilepsy. Though a significant proportion of patients did not display symptomatic improvement, clinical observational studies of the device showed a substantial reduction in seizure frequency [127,128]. Following the number of further clinical trials, observational studies and subsequent analysis led to the approval of VNS by the FDA in 2005 to treat pharmaco-resistant depression [129,130]. More recently, VNS has been evaluated and suggested as an alternative for treating a diverse array of disorders, including sepsis [131,132], heart failure [121,133], bowel disease [134], chronic pain [135], and rheumatoid arthritis inflammatory [136]. ElectroCore (Rockaway, NJ, USA) introduced GammaCore (nVNS), a noninvasive VNS, also referred to as the transcutaneous VNS (taVNS), is the most widely available device [137]. This handheld device could deliver alternating sinusoidal current with a broadband amplitude modulated frequency spectrum. In 2017 in USA., this device received FDA approval for treating acute cluster headache, and in 2018 the device received support for treating adjunctive cluster headache and acute migraine treatment [137]. Furthermore, NEMOS transcutaneous auricular vagus nerve stimulator (taVNS) is another one developed by Cerbomed GmbH, Erlangen, Germany. It is the most widely used commercially available taVNS device that could deliver current in rhythmic square pulses [138]. In 2010, this device received European certification for treating epilepsy and depression, in 2012 for chronic pain, and in 2019 for anxiety [138]. VNS devices developed companies are listed in Table 1. 

Although the peripheral and central nervous systems via electrical stimulation have attracted much attention due to their potential advantages on neuropsychiatric diseases, its non-cell type-specific activation characteristics could hinder its wide clinical applications. Therefore, optogenetic approaches have attracted more recently and been applied as a cell-specific approach to the vagus nerve for precise modulation of neural functions [139]. The commonly used optical waveguides are silica optical fibers that could help stimulate the vagus nerve optogenetically. Cao et al. successfully developed a simplified method for fabricating flexible and stretchable polymer optical fibers as optical waveguides [139]. Significant advantages of these fabricated polymer optical fibers are low young modulus, high stretchability, improved biocompatibility, and long-term stability. These important response characteristics suggest the optical fiber’s capability to implement in vivo optogenetic applications. To determine whether these polymer optical fibers could deliver enough light to modulate optogenetically in vivo, an adeno-associated virus was injected into the mouse hippocampus of mice. Subsequently, a custom-made opto-electrode array consisting of polymer optical fibers and two tetrodes was used to stimulate and record electrophysiological activities. Continuous frequency-dependent action potentials were detected with light pulses and delivered to the mice hippocampus, suggesting the fabricated polymer optical fibers can serve as waveguides for in vivo optogenetic stimulation. Further, the waveguides are implanted into the primary motor cortex to validate long-term stability. After four weeks of viral injection, blue light pulses were conducted through the implanted waveguides to activate the neurons. Continuous activations potentials of optically activated neurons were recorded. The recorded results suggest that these optical fibers as waveguides helped in the optogenetic stimulation treatment for improving the mice’s impairment [139]. Recently, a battery-free wireless-powered implantable high-performance hydrogel nanogenerator was developed by Chen et al. to stimulate the vagus nerve. The device works programmatically with the ultrasound pulses remotely. This novel triboelectric hydrogel nanogenerator was developed based on polyacrylamide/graphene conductive hydrogels [140]. The size of the device is 1.5 cm×1.5 cm×1 mm, the ultrasound power density of $0.3\;W/{\mathrm{cm}}^{2}$ delivers the short circuit current of about 1.6 mA, which is sufficient to stimulate the vagus nerve. Thus, a subcutaneously implanted device could directly serve as a wireless neurostimulator with the optimum current density and waveform programmed by external ultrasound pulses [140]. Eliminating the necessity of using rectifiers is a potential advantage of this device. Moreover, Zhang et al. developed a battery-free piezoelectric nanogenerator that could successfully harvest biomedical energy from the carotid artery pulsation to stimulate the vagus nerve. The piezoelectric nanogenerator was fabricated with the help of the electrospinning method [141]. This method helps produce layer-by-layer assembly using poly(vinylidene fluoride-co-trifluoroethylene) and barium titanium oxide. Thus, the significant advantage of this device has multi electricity generation units that constantly generate energy under even distribution of stresses. The fabricated device results showed induced power density is superior to that of most devices prepared with the same materials and is attributable to the even distribution of stresses on generating units. The stress mode that is evenly distributed inside a multi-unit generator was further conformed by COMSOL simulation analysis [141]. The device responses to the vagus nerve showed a decreased rate of heart canines [141], suggesting this could potentially stimulate the vagus nerve wirelessly without any complicated circuits and electrical components [141]. This kind of new generation of neurostimulation pathways with minimally invasive features could be a potential solution for the vagus nerve stimulation applications [140].

Gastric electrical stimulation has been an effective alternative to long-term dietary and medicinal treatment for gastroparesis and other stomach motility disorders. Stimulating stomach tissues could initiate neural activities in the vagus nerve is an alternative way for avoiding gastroparesis disorder [142]. Recently, Dubey et al. developed a miniaturized implantable battery-less gastro-stimulator with wireless power transfer capability [142]. The device harvests energy wirelessly and delivers the controlled electric pulses to the stomach tissues to initiate neural activities in vagus nerves and regain normal motility and contraction [142]. This device has novel wireless power transmitting antennae that deliver optimized power to generate controlled stimulative responses. Thus, the significant advantage of the developed vagus nerve stimulator. Furthermore, Wu et al. introduced power-efficient time to the current stimulator for vagus cardiac connection after heart transplantation [143]. This device aims to restore parasympathetic control after heart transplantation. The simulator is based on time to current conversion instead of conventional current mode digital to analog converter that drives the output current. It uses a digital to analog converter based on capacitor charging capability and operates as a power-efficient voltage to the current converter for the output. The simulator uses 1.8 V for system operation and 10 V for stimulation. The total power consumption of the device is low during the biphasic current out-put with a maximum I of 512 µA [143]. Moreover, Dabiri et al. developed a closed-loop system that behaves as a feedback system to reach optimum therapeutic outcomes [144]. A closed-loop feedback system is desirable to avoid over-stimulation and under-stimulation to the vagus nerve, leading to improved outcomes. Monitoring all significant physiological parameters and adjusting vagus nerve stimulation parameters according to the individual physiological data were used for designing a feedback loop. In addition, novel hardware was developed for the closed-loop system with diverse physiological sensing capability using external and/or embedded sensors [144]. The developed hardware processes and analyzed the acquired data in real-time and directly governs the stimulating parameters. Finally, Dabiri et al. showed aVNS stimulation device applicability with electrocardiography and photoplethysmography while mimicking baroreceptor-related afferent input along the afferent vagus nerve [144], and suggested the device that could identify >90% of cardiac cycles for activating the stimulation [144]. Vagus nerve stimulation devices with multi-functional advantages such as treatment for gastroparesis and other stomach motility disorders, vagus cardiac connection for cardiac disorders, devices with electronic feedback loops for gaining optimum outcomes [143], and with minimally invasive features could be a useful solution to overcome health disorders at single attempt rather than multiple surgeries and using multiple electroceuticals. However, any vagus nerve stimulation device’s efficiency depends on its performance of sub-electronic components. Among them, the amplifier’s efficiency plays a vital role [145]. Recently Devi et al. proposed the modified version of the cascaded recycling amplifier with the help of modelling and simulation tools [145]. The proposed design’s primary criterion is to use a preamplifier in an epileptic neural stimulator circuit with high gain, low noise, low power tradeoff, and smaller bandwidth (<500 Hz). The adaptive biasing technique was used to enhance the key parameters such as intrinsic gain, slew rate, and gain bandwidth for the proposed design. Finally, they showed the proposed design that can have the capability to work with ultra-low power consumption. The proposed method considers the high-frequency oscillations as a biomarker of focal epileptic seizures, owing to its occurrence in higher frequencies, thereby asserting a bandwidth less than 500 Hz. The simulation results showed improved parameters such as intrinsic DC gain of about 14 dB with a power consumption of 1.782 µW and the input noise of $10.97\;µV/\sqrt{\mathrm{Hz}}\;@1\;\mathrm{Hz}$ [145]. The feasibility of this kind of virtual concept from a concept to reality with the help of sophisticated fabrication technologies could greatly help us develop and use vagus nerve stimulation devices with multi-functional advantages for different health disorders in real-time.

Although multiple challenges and requirements concerning bioelectronic devices oppose the utilization of those in intelligent biological environments, several neurostimulators as vagus nerve stimulators are reported in the literature probably due to their potentiality towards multiple disorder treating capability and simplicity to adapt it to that of clinical conditions. Still, we need more realistic non-invasive vagus nerve stimulating devices for treating various biological diseases. Analytical modelling and simulation of vagus nerve stimulator design and comparative analysis with the commercially available VNS devices may help us to fabricate more reliable devices while enhancing miniaturization and ultra-low power consumption capability. We hope that the advancement in bioelectronics, with the help of allied areas [50,51], would help overcome the challenges and difficulties associated with traditional devices.

## 5. Safe Packaging against Electromagnetic Interference

Metals as shielding materials and packaging materials have become potential candidates for electroceuticals to avoid interference-induced abnormal conditions. The primary advantage of using metallic shielding is that it simply reflects the unwanted or disturbing electromagnetic fields. However, the biological interactions between the metallically shielded device and the surrounding biological tissues/media can cause the failure of shield, consequently, communication interruption or a device failure due to short circuits and loss of device’ properties [146,147,148]. Therefore, packaging must be biocompatible and electromagnetically compatible within the biological environment. Well-designed packaging within the availability of material resources is essential for those devices that must survive under biological environments. Hermetically compatible materials could be reliable candidates for implantable electroceuticals. Hermetically sealed metallic encapsulation is a significant protective layer and reflects the incident electromagnetic fields of all frequencies. Thus, there are no electromagnetic field interactions with the internal circuits/chips, no interference during operating conditions of the electroceutical device [146,147,148]. In addition to the significant shielding capability, it also exhibits biocompatibility and corrosion resistance behavior to sustain the electroceutical device within the biological environment.

## 6. Conclusions

Although this review article aims to introduce significant and potential development strategies of implantable biomedical electronics, their advancement, and materials, it is extremely difficult to cover all aspects due to the rapid development in this field. This article preferentially focuses on pacemaker devices, neural stimulation, artificial retina, and vagus nerve stimulation. Herein, we have reviewed the potential consequences of implantable electroceuticals. We have also summarized the risk factors and design challenges concerning the materials and biomedical device developments. We have also outlined the electromagnetic field influences on intelligent biomedical implants, their potential consequences, which would help design a reliable and qualified smart electroceutical implant for targeted clinical use. Finally, this review article highlights the importance of wirelessly supplying the necessary power and wirelessly triggering functional electronic circuits with ultra-low power consumption and multi-functional advantages such as monitoring and treating the disease in real-time.

## Figures and Tables

**Table 1:** World’s electroceutical device developing companies.

Retinal Prosthesis Devices	Implantable VNS Devices and External VNS Devices	Cardiac Pacemaker Devices	Deep Brain Stimulation Devices
Philips Healthcare Retina Implant Second Sight Medical Products Bionic Eye Technologies Bionic Vision Australia Vision Care Ophthalmic Tech. Abbott Laboratories	EnteroMedics, Inc. ElectroCore, Inc. Boston Scientific Corp. NeuroMetrix, Inc. Inspire Medical Systems, Inc. LivaNova, PLC. Electrocore, LLC. Parasym Ltd. Innovative Health Solutions tVNS Technologies GmbH	Medtronic, PLC. Boston Scientific Corp. Biotronik LivaNova, PLC. Abbott Laboratories Medico S.P.A. Shree Pacetronix Ltd. Osypka Medical, Inc. Lepu Medical Group	Medtronic plc Boston Scientific Corp. Abbott Laboratories Aleva Neurotherapeutics SA Deep Brain Innovations, LLC Beijing Pins Medical Co., Ltd.
